# The relationship between orthodontic treatment and temporomandibular disorders: A dental specialists’ perspective

**DOI:** 10.1590/2177-6709.27.1.e2220406.oar

**Published:** 2022-04-11

**Authors:** Dheaa H. AL-GROOSH, Mushriq ABID, Ahmed Kassem SALEH

**Affiliations:** 1 Department of Orthodontics, College of Dentistry/ University of Baghdad, Iraq.

**Keywords:** Orthodontics, Temporomandibular joint disorders, Perception

## Abstract

**Introduction:**

The relationship between temporomandibular disorders (TMDs) and orthodontic treatment/malocclusion has changed from a cause-and-effect association to an idea without sufficient evidence.

**Objective:**

This survey was designed to assess the beliefs of different disciplines - orthodontists, oral surgeons, and oral medicine specialists - on the relationship between TMDs and orthodontic treatment, with regard to treatment, prevention and etiology of TMDs.

**Method:**

A survey in the form of questionnaire was designed and distributed to 180 orthodontists, 193 oral surgeons and 125 oral medicine specialists actively involved in treating TMDs. The questionnaire aimed to collect basic information about each participant and their beliefs, and clinical management of patients with TMDs.

**Results:**

Halve of the responding orthodontists and most of the oral surgeons (69.9%) were male participants, whereas the majority of oral medicine specialists (83.3%) were female respondents. The participants’ age ranged from 29 to 58 years. The majority of orthodontists believes that there is no relationship between orthodontic treatment and TMDs, and that orthodontic treatment does not provoke TMDs or prevent the onset of the disorder. However, oral surgeons and oral medicine specialists have different and conflicting opinions. Most surgeons tended to treat those patients, while most of the other two disciplines tended to seek an interdisciplinary approach. Chi-square test was done to find an association between the referral status and specialists’ experience, and to compare between the different disciplines’ belief.

**Conclusions:**

Orthodontist’s beliefs were in accordance with the scientific evidence, whereas most oral surgeons and oral medicine specialist believed that orthodontic treatment may provoke TMDs. Therefore, continuing program series development is important to embrace the concept of the multidisciplinary team approach and improve the health care and quality of life for those patients.

## RESUMO

**Introdução:**

A relação entre as disfunções temporomandibulares (DTMs) e o tratamento ortodôntico/más oclusões teve como base uma associação de causa e efeito, e acabou migrando para um conceito sem evidência suficiente.

**Objetivo:**

A presente pesquisa foi desenhada para avaliar as crenças de diferentes especialidades - ortodontistas, cirurgiões bucomaxilofaciais e especialistas em Medicina Oral -, sobre a relação entre DTMs e a terapia ortodôntica, quanto ao tratamento, prevenção e etiologia das DTMs.

**Métodos:**

Foi feito um levantamento em formato de questionário, enviado para ortodontistas, cirurgiões bucomaxilofaciais e especialistas em Medicina Oral (180, 193 e 125, respectivamente) envolvidos ativamente no tratamento de DTMs. O questionário teve como objetivo coletar informações básicas sobre cada profissional, suas crenças e a conduta clínica em pacientes com DTM.

**Resultados:**

Metade dos ortodontistas que responderam e a maioria dos cirurgiões bucomaxilofaciais (69,9%) eram homens, enquanto a maioria dos especialistas em Medicina Oral (83,3%) era de mulheres. A idade dos participantes variou de 29 a 58 anos. A maioria dos ortodontistas acredita que não há relação entre tratamento ortodôntico e DTMs, e que o tratamento ortodôntico não provoca DTMs ou evita o aparecimento da disfunção. No entanto, cirurgiões bucomaxilofaciais e especialistas em Medicina Oral apresentam ideias diferentes e conflitantes. A maioria dos cirurgiões tendeu a tratar esses pacientes, enquanto a maioria das outras especialidades apresentou a tendência de buscar uma abordagem multidisciplinar. O teste do qui-quadrado foi realizado para encontrar uma associação entre a quantidade de encaminhamentos e a experiência dos especialistas, e para realizar uma comparação entre as crenças das diferentes especialidades.

**Conclusões:**

A crença dos ortodontistas esteve em acordo com a evidência científica, enquanto a maioria dos cirurgiões e dos especialistas em Medicina Oral acredita que o tratamento ortodôntico pode provocar DTMs. Dessa forma, o desenvolvimento de programas de educação continuada é importante para reforçar a necessidade da abordagem multidisciplinar e melhorar os cuidados orais e a qualidade de vida desses pacientes.

## INTRODUCTION

Temporomandibular disorders (TMDs) are a group of neuromuscular and musculoskeletal conditions involving the masticatory muscles, temporomandibular joint complex and the surrounding bony structures. TMDs are a multifactorial condition with psychogenic influence of different degrees, affecting an individual’s quality of life. Several etiological factors were believed to cause the disorder such as trauma, underlying occlusal anomalies and emotional stress.^1^ ^,^ ^2^ The disorder is not uncommon and affects about 26 to 46% of young adults.^3^ ^,^ ^4^ Common symptoms of TMDs include headache, facial pain, jaw dysfunction and TM pain.^5^ ^,^ ^6^ It was believed that there is similarity between TMDs pain and lower back pain, considering individual variation in pain perception. Since there is a strong relationship between TMDs and occlusion, changing the position of teeth and altering the existing occlusion via orthodontic appliances has drawn many investigations and controversial opinions, without establishing a conclusive evidence.^7^

In the last decade, researchers conducted several studies to explain the relationship between TMDs and orthodontic treatment.^8^ ^,^ ^9^ Despite the use of sophisticated and modern diagnostic tools such as magnetic resonance imaging, and scientific studies with long-term follow-up, it has not yet been possible to eliminate this existing controversy.^10^ Although there is no significant evidence supporting the predisposing effect of orthodontic treatment on TMDs and occlusion, the treatment is not indicated as a therapeutic measure or a means to decrease the risk of the disorders.^11^ ^,^ ^12^ However, the attention given to signs and symptoms associated with TMDs has modified the clinical management before and during orthodontic treatment.^13^

Although the disorder has a normal cycle of events, appearing to spontaneously improve without treatment, the treatment of such a group of disorders involves a multidisciplinary approach with robust protocols.^14^ ^,^ ^15^ A team of oral medicine specialists, surgeons and orthodontist may collaborate to manage these disorder using protocols ranging from nonsurgical medications to surgical interventions. 

To the authors’ knowledge, the perception of orthodontists, oral surgeons and the oral medicine specialists together was not investigated; therefore, this study was designed to assess the beliefs, despite scientific evidence, of the TMDs team of specialists about the relationship between TMDs and orthodontic treatment, with regard to treatment, prevention and possible etiology.

## MATERIAL AND METHODS

The study was approved by the scientific research and ethics committee of College of Dentistry, University of Baghdad (Approval no. 361/ 2019). A questionnaire was modified from Coêlho and Caracas^16^ and sent to dental specialists actively involved in treating temporomandibular disorders. The questionnaire was distributed to Iraqi orthodontists, oral surgeons and oral medicine specialists nationwide (180, 193 and 125 respectively; with an age range of 29-58 years) via e-mail to their corresponding professional societies. The e-mail explained the survey objectives and contained a link to direct the respondents to the website where the questionnaire could be answered (via Google forms). Some specialists were not members of their professional societies and have been approached through their correspondent groups, using social network apps, where the online questionnaire link was attached. Each participant accessed the questionnaire via his/her official login account information, and was allowed to answer the questions once. To avoid non-specialists or specialists who have not treated patients with TMDs from participating, obligatory filter questions were designed and applied as a mandatory task before start answering the questionnaire. These questions were related to the participant’s discipline and whether they have treated patients with TMDs, as follows: *“Are you a specialist of one of the following disciplines: Orthodontics, Oral Surgery or Oral Medicine?”* and, *“Have you treated patients with TMDs?”*. These filter questions disabled respondent who answered “No” from participating.

The survey took ten weeks, from December 2019 to February 2020, and the participants were advised to contact the authors for inquiries related to answering the questionnaire. The questionnaire consisted of questions related to participants’ basic information and others to collect information about beliefs and clinical protocols while treating patients with TMDs. 

**Figure 1:**
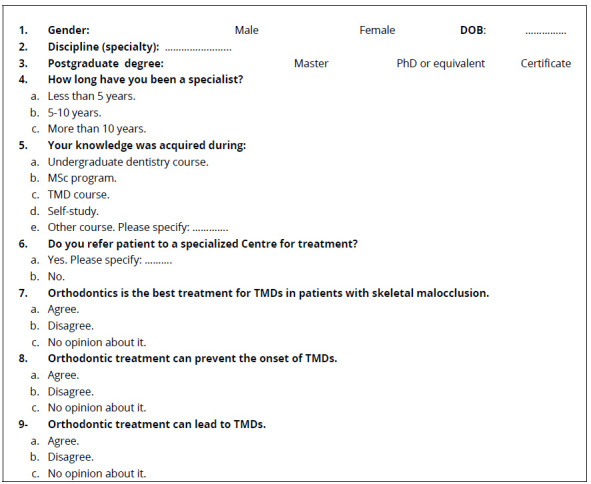
Sample of the survey questionnaire.

The survey included questions related to participant’s general information: gender, discipline, clinical professionalism and the source of TMDs knowledge. In addition, the survey included questions related to specialists’ belief regarding the relationship between orthodontic treatment and TMDs, i.e. whether orthodontic treatment can treat, prevent or lead to TMDs. A sample of the survey questionnaire is presented in the Figure 1. 

A pilot study was conducted on 15 academics and experienced specialists (five from each discipline), using the same questionnaire, to minimize unclear and ambiguous questions. Consequently, the questions were reviewed and modified to ensure scientific accuracy. 

The sample size was calculated using the following formulae: ^17^

(1) $$Required\;sample\;size\;=\;p\left(100–p\right)z^{2}/E^{2}$$

(2) $$True\;sample\;=\;\frac{\left(required\;sample\;size\;x\;population\right)}{\left(required\;sample\;size\;+\;population\;–1\right)}$$

Where ‘p’ is 50% of a sample; ‘z’ is the level of confidence, which is equal to 1.96 (for confidence level of 95%), and ‘E’ is the margin of error = 0.05.

After data acquisition, descriptive statistic represented by tables and histograms was used to analyze the percentages of the respondents’ answers. Additionally, chi-square test was done to find an association between the referral status and specialists’ experience, and to compare between the different disciplines’ belief.

## RESULTS

The results of the current study revealed that there was a good rate of participation from the specialists: 112 orthodontists (62.2%), 136 oral surgeons (71.5%) and 84 oral medicine specialists (67.2%). Almost half of the responding orthodontists (47.9%) have less than five years’ experience, while 23.9% have more than 10 year experience. Nearly 40% of oral surgeons and oral medicine specialists have more than 10 years’ experience (Table 1). Halve of the responding orthodontists and the majority of the oral surgeons (69.9%) were male participants, whereas the majority of oral medicine specialists were female respondents (83.3%).

**Table 1:** Response rate and cumulative experience.

Participants	Response rate	Gender distribution		Cumulative experience		
		Male	Female	Less than 5 years	5-10 years	More than 10 years
Orthodontists n (%)	112 (62.2 %)	56 (50%)	56 (50%)	54 (47.9%)	30 (28.2%)	28 (23.9%)
Oral surgeon n (%)	136 (71.5 %)	95 (69.9%)	41 (30.1%)	48 (35.3%)	34 (25%)	54 (39.7%)
Oral medicine n (%)	84 (67.2 %)	14 (16.7%)	70 (83.3%)	28 (33.3%)	22 (26.2%)	34 (40.5%)

**Figure 2:**
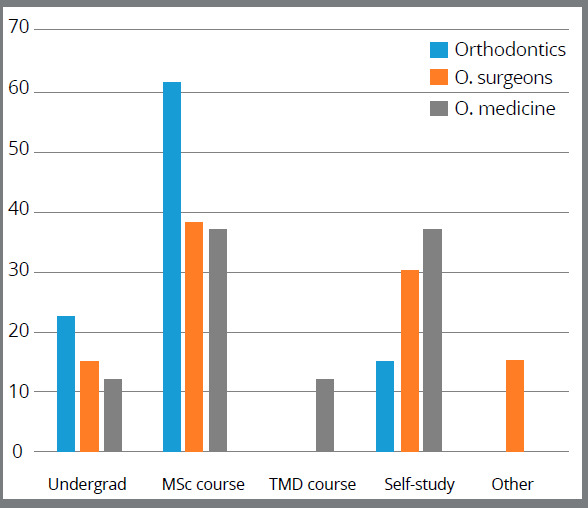
Source of knowledge, with regards to disciplines.

The majority of the participants achieved their TMD knowledge from their postgraduate MSc program, in addition to their basic undergraduate program, specially the orthodontists (61.5% and 23.1%, respectively). Self-learning activity accounted for 15.4% of the orthodontists, who exhibited the least percentage compared to oral surgeons (30.8%) and oral medicine specialists (37.5%). Only a small proportion of the oral medicine specialists (12.5%) attended specialized TMD courses, as shown in Figure 2.

There was a statistically significant association between the time after training program and the referral status of the orthodontists and the oral surgeons (*p*< 0.001). Less time since the training program was related to more referral status for orthodontists, however, the opposite was true for the oral surgeons, as shown in Table 2. The majority of oral surgeons (77%) answered no for the question related to interdisciplinary cooperation. On the other hand, orthodontists and oral medicine specialists tended to refer the patient for further consultations in a percentage of 62.5% and 50% respectively. Indeed, the oral medicine specialists had an equal response.

**Table 2:** Interdisciplinary referral data and its relationship to the time after specialist training program.

Health professionals	Referral status	n (%)	Cumulative experience (n (%))			Chi-square	p-value
			Less than 5 years	6-10 years	More than 10 years		
Orthodontist	Refer	70 (62.5)	44 (39.2)	16 (14.2)	10 (9)	17.948	0.000
	Not refer	42 (37.5)	10 (9)	14 (12.6)	18 (16)		
	Total	112 (100)	54 (48.2)	30 (26.8)	28 (25)		
O. surgeon	Refer	30 (22.2)	19 (14)	5 (3.7)	6 (4.4)	13.408	0.001
	Not refer	106 (77.8)	29 (21.3)	29 (21.3)	48 (35.3)		
	Total	136 (100)	48 (35.3)	34 (25)	54 (39.7)		
O. medicine	Refer	42 (50)	17 (20.2)	9 (10.7)	16 (19.1)	2.131	0.344
	Not refer	42 (50)	11 (13.1)	13 (15.5)	18 (21.4)		
	Total	84 (100)	28 (33.3)	22 (26.2)	34 (40.5)		

Table 3 shows that the participating health professionals had statistically significant different opinions regarding the relationship between orthodontics, as treatment or preventive means, and TMDs (*p*= 0.001). The majority of the oral surgeons and oral medicine specialists (70% and 83%, respectively) believed that orthodontic treatment is the best option for TMDs patients; additionally, they believed that it prevents the onset of the disorder. Contrary to that, most of the orthodontists (87.5%) disagreed or didn’t have an opinion regarding the role of orthodontic therapy on TMDs treatment. Moreover, half of the orthodontists disagreed or had no opinion regarding the role of orthodontic treatment in preventing TMDs. 

**Table 3:** Comparison among specialists’ responses on whether orthodontic treatment can treat or prevent TMD problems.

Orthodontic treatment is best treatment for TMDs						
Health professionals	Agree	Disagree	No opinion	Total	Chi-square	
	n (%)	n (%)	n (%)	n (%)	df	p-value
Orthodontist	14 (12.5)	42 (37.5)	56 (50)	112 (100)	4	0.001
O. Surgeon	95 (70)	0 (0)	41 (30)	136 (100)		
O. Medicine	70 (83.3)	0 (0)	14 (16.7)	84 (100)		
Total	179 (53)	42 (12.6)	111(33.4)	332 (100)		
Orthodontic treatment can prevent the onset of TMDs						
Health professionals	Agree	Disagree	No opinion	Total	Chi-square	
	n (%)	n (%)	n (%)	n (%)	df	p-value
Orthodontist	56 (50)	28 (25)	28 (25)	112 (100)	4	0.001
O. surgeon	68 (50)	42 (30)	26 (20)	139 (100)		
O. medicine	70 (83.3)	14 (16.7)	0 (0)	84 (100)		
Total	194 (58.4)	84 (25.3)	54 (16.3)	332 (100%)		

Table 4 reveals that there was a significant difference regarding answers about whether orthodontic treatment can cause TMDs (*p*= 0.001). More than two-thirds of the oral medicine specialists (66.7%) and half of the oral surgeons (50%) agreed or had no opinion that orthodontic treatment can lead to TMDs problems; whereas most of the orthodontists disagreed with this claim (75%). 

**Table 4:** Comparison among specialists’ responses on whether orthodontic treatment can lead to TMDs problems.

Orthodontic treatment can lead to TMDs						
Health professionals	Agree n (%)	Disagree n (%)	No opinion n (%)	Total n (%)	Chi-square	
					df	p-value
Orthodontist	28 (25)	84 (75)	0 (0)	112 (100)	4	< 0.001
O. surgeon	68 (50)	27 (20)	41 (30)	136 (100)		
O. medicine	56 (66.7)	0 (0)	28 (33.3)	84 (100)		
Total	152 (45.8)	111 (33.5)	69 (20.7)	332 (100)		

## DISCUSSION

Although the use of questionnaires is fundamental for knowledge and perceptive research, it may have some limitations, such as poor adhesion of participants, which reduces the number of answers, low response rate and, sometimes inconsistency of the answers.^18^ The authors tried to reduce these issues by using the web-based questionnaire,^19^ however, online survey may discriminate senior individuals who are not regular ‘cyber users’, which may, in turn, affect the overall data. This comes in agreement with a previous study reporting that the age of participants may affect their willingness to participate, especially in web-based questionnaire.^20^ In this context, the sample size of the current study was in accordance with the calculated sample size, which yields 95% confidence interval (117, 132 and 82 for orthodontists, oral surgeons and oral medicine specialists respectively). Another limitation of the current questionnaire is the use of an e-mail as a mean to send the survey. This may have brought a risk of having non-specialists or specialists who are not treating patients with TMDs participating. However, the authors overcame this issue by asking the participants to answer obligatory filter questions about the type of specialist training program and whether the participant treated patients with TMDs or not. Therefore, respondents who have not fulfilled the required profession or those who have not treated patients with TMDs were unable to continue answering the questions (due to the nature of the web-based questionnaire). Yet, misreporting these questions may be encountered. 

The results of the current survey showed that the response rate was higher than in the previous survey conducted on orthodontists.^16^ This finding is in accordance with Saleh and Bista,^20^ who reported that the participation rate was higher in online based surveys, compared to the conventional ones. This could be due to several influencing factors such as survey structure, communication methods, professionalism (target group) and simplicity of the questions. The setup of questions and question-answering process in online based questionnaire made handling the survey questions an easy task.^20^

The majority of orthodontists believed that orthodontic treatment had no effect on TMDs symptoms. This is in accordance with the scientific evidence in which most of the previous studies suggested that orthodontic treatment neither prevents nor causes TMDs.^21^ ^-^ ^23^ However, oral surgeons and oral medicine specialists opposed this opinion, which disagreed with the findings reported by Leite et al,^24^ who suggested that orthodontic treatment did not provoke the risk of developing signs and symptoms of TMDs, regardless the technique used for treatment and the extraction status.

Most of the orthodontists and a few oral surgeons agreed that orthodontic treatment has no effect on or prevents TMDs, which is in accordance with other studies.^16^ ^,^ ^24^ The concept of orthodontic treatment as a choice to solve TMDs symptoms may be differently interpreted among disciplines. Both oral surgeons and oral medicine specialists interpreted orthodontic treatment as a mean to solve the malocclusion, which was considered as a predisposing factor to TMDs. This contradicted the findings of previous studies reporting that the majority of the published articles failed to identify any significant and clinically important associations between certain type of malocclusions and TMDs.^25^ ^,^ ^26^ TMDs could not be correlated to any specific type of malocclusion, and there was no support for the belief that orthodontic treatment may cause TMDs. Obvious individual variations in signs and symptoms of TMDs over time, according to some longitudinal studies, emphasized the difficulty in establishing malocclusion as a significant risk factor for TMDs.^27^ ^,^ ^28^ This belief comes in parallel with the referral profile of the orthodontists, as they believed that causes other than malocclusion may be responsible for TMDs risk and severity. 

The questionnaire outcomes suggested a lack of clear clinical guidance and evaluating protocols determining the role of different health professionals in management of patients with TMDs. Additionally, despite of professionals’ competence in treatment of TMDs, patients may be undertreated or under-evaluated. This may be due to lack of awareness to the possible therapeutic options currently available and lack of interdisciplinary approach to exchange knowledge and clinical experience through joint meeting.^29^ ^,^ ^30^

Therefore, setting up interdisciplinary clinics, with oral surgeons, oral medicine specialists and orthodontists involved in a coordinated way with different treatment approaches, is recommended. Moreover, continuing program development series is important to embrace the concept of the multidisciplinary team approach and improve the health care service and the quality of life for those patients. 

## CONCLUSIONS

The majority of orthodontists’ beliefs came in accordance with the scientific evidence regarding the lack of relationship between orthodontic treatment and TMDs symptoms, and that orthodontic treatment does not necessarily prevent the onset of TMDs. This was significantly different from the point of views of other participating health professionals, who believed that orthodontic treatment has implications to TMDs. The study, additionally, showed that there is a statistically significant association between the time after specialists’ training program and the referral status. The majority of the oral surgeon tried to treat those patients, unlike the orthodontists and the oral medicine specialists, who seemed to have an interdisciplinary approach attitude. 

## References

[1] Sonnesen L, Bakke M, Solow B (2001). Temporomandibular disorders in relation to craniofacial dimensions, head posture and bite force in children selected for orthodontic treatment. Eur J Orthod.

[2] Suvinen TI, Reade PC, Kemppainen P, Könönen M, Dworkin SF (2005). Review of aetiological concepts of temporomandibular pain disorders Towards a biopsychosocial model for integration of physical disorder factors with psychological and psychosocial illness impact factors. Eur J Pain.

[3] Casanova-Rosado JF, Medina-Solís CE, Vallejos-Sánchez AA, Casanova-Rosado AJ, Hernández-Prado B, Ávila-Burgos L (2006). Prevalence and associated factors for temporomandibular disorders in a group of Mexican adolescents and youth adults. Clin Oral Investig.

[4] Loster JE, Osiewicz MA, Groch M, Ryniewicz W, Wieczorek A (2017). The Prevalence of TMD in Polish Young Adults. J Prosthodont.

[5] Motghare V, Kumar J, Shivalingesh KK, Kushwaha S, Anand R, Gupta B (2015). Association between harmful oral habits and sign and symptoms of temporomandibular joint disorders among adolescents. J Clin Diagnostic Res.

[6] Gauer RL, Semidey MJ (2015). Diagnosis and treatment of temporomandibular disorders. Am Fam Physician.

[7] Thilander B, Rubio G, Pena L, Mayorga C (2002). Prevalence of temporomandibular dysfunction and its association with malocclusion in children and adolescents An epidemiologic study related to specified stages of dental development. Angle Orthod.

[8] Manfredini D, Stellini E, Gracco A, Lombardo L, Nardini LG, Siciliani G (2016). Orthodontics is temporomandibular disorder-neutral. Angle Orthod.

[9] Fernández-González FJ, Cañigral A, López-Caballo JL, Brizuela A, Moreno-Hay I, Río-Highsmith JD (2015). Influence of orthodontic treatment on temporomandibular disorders A systematic review. J Clin Exp Dent.

[10] Conti A, Freitas M, Conti P, Henriques J, Janson G (2003). Relationship between signs and symptoms of temporomandibular disorders and orthodontic treatment A cross-sectional study. Angle Orthod.

[11] Carrara SV, Rodrigues Conti PC, Barbosa JS (2010). Statement of the 1st Consensus on Temporomandibular Disorders and Orofacial Pain. Dental Press J Orthod.

[12] Sim HY, Kim HS, Jung DU, Lee H, Han YS, Han K (2019). Investigation of the association between orthodontic treatment and temporomandibular joint pain and dysfunction in the South Korean population. Korean J Orthod.

[13] Giray B, Sadry S (2021). Modifications in Class I and Class II Div 1 malocclusion during orthodontic treatment and their association with TMD problems. Cranio.

[14] Litt MD, Shafer DM, Kreutzer DL (2010). Brief Cognitive-Behavioral Treatment for TMD Pain Long-Term Outcomes and Moderators of Treatment. Pain.

[15] Manfredini D, Piccotti F, Guarda-Nardini L (2010). Hyaluronic acid in the treatment of TMJ disorders A systematic review of the literature. Cranio.

[16] Coêlho TGS, Caracas HCPM (2015). Perception of the relationship between TMD and orthodontic treatment among orthodontists. Dental Press J Orthod.

[17] Taherdoost H (2017). Determining sample size; How to calculate survey sample size. International Journal Economics Management Systems.

[18] Martins RL, Kerber FC, Stuginski-Barbosa J (2011). Attitudes of a group of Brazilian orthodontists towards the diagnosis and management of primary headache (migraine) An electronic-based survey. J Appl Oral Sci.

[19] Ebert JF, Huibers L, Christensen B, Christensen MB (2018). Paper- or Web-Based Questionnaire Invitations as a Method for Data Collection Cross-Sectional Comparative Study of Differences in Response Rate, Completeness of Data, and Financial Cost. J Med Internet Res.

[20] Saleh A, Bista K (2017). Examining factors impacting online survey response rates in educational research perceptions of graduate students. J Multi Discip Eval.

[21] Luther F (1998). Orthodontics and the temporomandibular joint Where are we now? Part 1. Orthodontic treatment and temporomandibular disorders. Angle Orthod.

[22] Ortega ACBA, Pozza DH, Rodrigues LLFR, Guimarães AS (2016). Relationship Between Orthodontics and Temporomandibular Disorders A Prospective Study. J Oral Facial Pain Headache.

[23] Tagkli A, Paschalidi PP, Katsadouris A, Tsolakis A (2017). Relationship between Orthodontics and Temporomandibular Disorders. Balk J Den Med.

[24] Leite RA, Rodrigues JF, Sakima MT, Sakima T (2013). Relationship between temporomandibular disorders and orthodontic treatment A literature review. Dental Press J Orthod.

[25] Rendell JK, Norton LA, Gay T (1992). Orthodontic treatment and temporomandibular joint disorders. Am J Orthod Dentofac Orthop.

[26] Egermark I, Carlsson GE, Magnusson T (2005). A prospective long-term study of signs and symptoms of temporomandibular disorders in patients who received orthodontic treatment in childhood. Angle Orthod.

[27] Sadowsky C, Theisen TA, Sakols EI (1991). Orthodontic treatment and temporomandibular joint sounds--A longitudinal study. Am J Orthod Dentofac Orthop.

[28] Hirata RH, Heft MW, Hernandez B, King GJ (1992). Longitudinal study of signs of temporomandibular disorders (TMD) in orthodontically treated and nontreated groups. Am J Orthod Dentofac Orthop.

[29] Greene CS, Galang-Boquiren MTS, Bartilotta BY (2017). Orthodontics and the Temporomandibular Joint what orthodontic providers need to know. Quintessence Int.

[30] Correia LMF, Silva JW, Lima HLC, Krakauer M (2019). Interdisciplinary care in the treatment of orofacial pain Case report. BrJP.

